# Hydroxychloroquine suppresses replication of PRRSV via dual regulation of autophagic flux and NF-κB-driven inflammatory signaling

**DOI:** 10.1186/s13567-026-01855-2

**Published:** 2026-10-27

**Authors:** Ruirui Ye, Pengzhan Yuan, Guoxin Zheng, Bin Kang, Haijun Guo, Yankuo Sun, Guihong Zhang, Heng Wang

**Affiliations:** 1 Guangdong Provincial Key Laboratory of Zoonosis Prevention and Control, College of Veterinary Medicine, South China Agricultural University, Guangzhou, 510462 China; 2 National Engineering Research Center for Breeding Swine Industry, South China Agricultural University, Guangzhou, 510642 China; 3 Hangzhou Uben Animal Vaccine Co., Ltd, Hangzhou, 310000 China

**Keywords:** Hydroxychloroquine, porcine reproductive and respiratory syndrome virus (PRRSV), autophagic flux, NF-κB signaling, broad-spectrum antiviral activity, swine RNA viruses

## Abstract

Porcine reproductive and respiratory syndrome virus (PRRSV) induces excessive autophagy and aberrant nuclear factor kappa-light-chain-enhancer of activated B cell (NF-κB) hyperactivation, triggering severe respiratory and reproductive disorders in pigs and causing massive economic losses to the global swine industry. Hydroxychloroquine sulfate (HCQ), a classic autophagy inhibitor, restricts PRRSV proliferation in vitro; however, its in vivo protective efficacy and dual-targeted molecular mechanisms remain poorly characterized. In this study, intramuscular HCQ administration conferred robust protection against lethal PRRSV infection in piglets: it completely eliminated virus-induced mortality, relieved fever and interstitial pulmonary lesions, and restored normal growth performance. In vitro assays confirmed that HCQ with negligible cytotoxicity within the concentration range of 1–100 μM interferes with multiple stages of PRRSV infection. Combined network pharmacology and cellular functional experiments revealed a dual regulatory mechanism of HCQ against PRRSV: HCQ blocks autophagosome–lysosome fusion to disrupt complete autophagic flux, and suppresses the IκBα-p65 signaling cascade to reduce the secretion of proinflammatory mediators. Dose–response analyses demonstrated that HCQ exerts potent antiviral activity against divergent field PRRSV isolates, porcine epidemic diarrhea virus (PEDV) and swine acute diarrhea syndrome coronavirus (SADS-CoV) within a wide safe therapeutic window, with all broad-spectrum in vitro antiviral activity against diverse swine RNA viruses validated in cell culture models. Collectively, HCQ suppresses PRRSV replication by simultaneously regulating autophagic flux and NF-κB-dependent inflammatory signaling, supporting its repurposing as a promising therapeutic candidate for porcine reproductive and respiratory syndrome (PRRS) control. Although HCQ exhibits broad-spectrum inhibitory activity against divergent PRRSV strains, PEDV, and SADS-CoV in cell culture, all anti-PEDV and anti-SADS-CoV effects are only validated in vitro without in vivo animal verification.

## Introduction

Porcine reproductive and respiratory syndrome virus (PRRSV) causes an acute, highly contagious viral disease that affects swine populations and is characterized by reproductive disorders and respiratory symptoms (porcine reproductive and respiratory syndrome [PRRS]) [1]. PRRSV is a single-stranded, positive-sense RNA virus (internal nucleocapsid diameter, approximately 25–30 nm) belonging to the genus *Arterivirus* of the family *Arteriviridae* [2]. As a positive-sense single-stranded RNA arterivirus, PRRSV is classified into European PRRSV-1 and North American PRRSV-2 genotypes with substantial genomic divergence [3]. The full-length PRRSV genomic RNA is approximately 15.4 kb, encoding an array of structural and nonstructural proteins that coordinate viral immune evasion and persistent infection [4, 5].

PRRSV utilizes diverse molecular mechanisms to antagonize host innate immune surveillance, disrupting inflammatory homeostasis and increasing susceptibility to secondary bacterial coinfections [6]. Since its emergence over three decades ago, PRRS has caused devastating economic losses to global pig production through elevated mortality and costly epidemic management. Current commercial vaccines only provide limited cross-protection against continuously mutated field PRRSV strains, while effective broad-spectrum small-molecule antivirals for swine RNA viruses remain scarce, creating an unmet demand for systematically characterized host-targeted therapeutic candidates.

Hydroxychloroquine sulfate (HCQ), a low-toxicity derivative of chloroquine, is a World Health Organization (WHO) essential medicine widely applied against malaria and chronic inflammatory disorders [7, 8]. As a weak lysosomotropic base, HCQ accumulates within lysosomes to neutralize luminal acidity, suppress lysosomal hydrolase activity, and ultimately block autophagosome–lysosome fusion to interrupt complete autophagic flux. Compared with chloroquine, HCQ exhibits superior tissue tolerance and lower systemic toxicity, making it more suitable for in vivo antiviral intervention [9–11]. Accumulating evidence indicates that HCQ alleviates virus-induced tissue injury by inhibiting excessive autophagy and overactivated inflammatory signaling [12]. Many economically important swine RNA viruses hijack host autophagic machinery to promote replication and drive pathological inflammation; therefore, targeting the autophagy–inflammation axis via HCQ represents a promising universal antiviral strategy for swine viral diseases [13–15].

Autophagy is an evolutionarily conserved intracellular degradation and recycling pathway that maintains cellular homeostasis and participates in host defense against invading pathogens [16]. Abundant studies have validated that PRRSV actively induces functional autophagic flux to boost its replication [17, 18]. The autophagy activator rapamycin increases PRRSV yields, whereas autophagy inhibitors 3-methyladenine and bafilomycin A1 markedly reduce viral loads, demonstrating that PRRSV replication relies on intact autophagic machinery [19–21]. Autophagy is tightly coupled with inflammatory signaling, and PRRSV rewires autophagic cascades to facilitate immune evasion [22]. Recent mechanistic research reveals that PRRSV manipulates MALT1-dependent autophagic membrane remodeling, and viral GP5 protein blocks chaperone-mediated antiviral autophagy by targeting LAMP2A [23]. Elucidating the reciprocal interaction between PRRSV and autophagy helps uncover viral pathogenic mechanisms and facilitates the development of systematically characterized anti-PRRSV therapies [24]. Numerous swine RNA viruses hijack host autophagy to facilitate propagation; accordingly, pharmacological autophagy suppression represents a feasible broad-spectrum antiviral strategy [25]. We thus characterized whether HCQ exerts panviral activity against viruses other than PRRSV. Collectively, pharmacological modulation of autophagy provides a promising strategy to restrain PRRSV and combat diverse swine viral pathogens.

This study systematically investigates the in vitro and in vivo anti-PRRSV potency of HCQ, dissects its inhibitory regulation on autophagy and NF-κB-mediated inflammatory signaling upon PRRSV infection, and further evaluates its broad-spectrum antiviral activity against other swine RNA viruses. These findings provide experimental and theoretical evidence for repurposing HCQ as a therapeutic candidate against PRRS and other swine viral diseases.

## Materials and methods

### Cell culture, strains, and reagents

Porcine alveolar macrophages (PAMs) and Marc-145 cells, routinely used for PRRSV propagation, were preserved in the College of Veterinary Medicine, South China Agricultural University. PAMs were isolated from the lungs of 28-day-old healthy piglets via bronchial lavage. Briefly, lungs were repeatedly perfused with precooled sterile phosphate-buffered saline (PBS), cell pellets were harvested by centrifugation, and red blood cells were eliminated using erythrocyte lysis buffer. Purified PAMs were resuspended in cryopreservation medium and stored in liquid nitrogen. Before each experiment, frozen PAMs were thawed, recovered, and cultured for 12 h to stabilize cellular morphology and activity prior to seeding.

Cells were cultured in complete medium supplemented with 10% fetal bovine serum (Gibco, USA), 100 U/mL penicillin, and 100 μg/mL streptomycin. PRRSV strains XH-GD, GM30, and NADC30, and porcine epidemic diarrhea virus (PEDV) strain FS202201 were maintained in our laboratory. XH-GD (GenBank: EU624117.1) is a JXA1-like highly pathogenic PRRSV-2 strain isolated from pig farms in Guangdong Province. GM30 is a circulating NADC30-like recombinant strain prevalent in South China with moderate virulence. NADC30 (GenBank: JN654459.1) is the prototype NADC30-origin strain with moderate pathogenicity, carrying a characteristic large deletion in the nonstructural protein 2 (*nsp2*) gene. The PEDV strain FS202201 belongs to the highly virulent G2c variant subtype, isolated from diarrheal piglet clinical samples in 2022. Swine acute diarrhea syndrome coronavirus (SADS-CoV) strain GDS04_P12 was generously provided by Prof. Yongchang Cao (School of Life Sciences, Sun Yat-sen University) and preserved in Prof. Guihong Zhang’s laboratory. GDS04_P12 is a Vero cell-adapted serial passage strain derived from wild isolate GDS04 (GenBank: MF167434.1); the original wild strain shows high virulence to suckling piglets, while the P12 cell-adapted variant exhibits attenuated replication capacity in vitro. The Cell Counting Kit-8 (CCK-8) was purchased from Beyotime Biotechnology (Shanghai, China).

### Chemicals and antibodies

Primary antibodies used in this study were as follows: LC3-II (ab192890, Abcam), HA (3724S, Cell Signaling Technology), Flag (AF519, Beyotime), GAPDH (HC301, TransGen Biotech), SQSTM1/p62 (A19700, ABclonal), β-tubulin (GTX101279, GeneTex), NF-κB p65 (8242 T, Cell Signaling Technology), phospho-IκBα (Ser32) (2859, Cell Signaling Technology), and total IκBα (4812, Cell Signaling Technology). Fluorescent secondary antibodies conjugated to Alexa Fluor 488, 594, and 647 were obtained from Invitrogen (Carlsbad, CA, USA). HRP-conjugated anti-mouse and anti-rabbit immunoglobulin G (IgG) secondary antibodies were supplied by Beyotime. Hydroxychloroquine sulfate (HCQ, Cat. T0951, Targetmol, Shanghai, China, HPLC purity = 98.92%) was used in this study. Homemade hybridoma supernatants containing monoclonal antibodies against PRRSV nucleoprotein (N), PEDV N, and SADS-CoV N were used for immunofluorescence and western blot detection, and all antibody stocks were long-term preserved in our laboratory.

### Cell viability assays

Marc-145 or PAM cells were seeded into 96-well plates at the identical density of 4 × 10^4^ cells per well and incubated at 37 °C for 12 h prior to HCQ treatment. Cells were then treated with serially diluted HCQ (1–1500 μM), with eight biological replicates per concentration. After 36 h of incubation, 10 μL CCK-8 solution was added to each well, followed by a 1 h incubation at 37 °C. The optical density (OD) value at 450 nm was detected using a microplate reader (Thermo Fisher Scientific).

### Western blotting

Cells were lysed with radioimmunoprecipitation assay (RIPA) lysis buffer (Beyotime, P0013) to extract total cellular protein. Protein samples were separated by sodium dodecyl sulfate–polyacrylamide gel electrophoresis (SDS-PAGE) and transferred onto nitrocellulose membranes (Millipore). After blocking with 5% skim milk, membranes were incubated with corresponding primary antibodies overnight at 4 °C, followed by 1 h incubation with secondary antibodies at 37 °C. Target protein bands were detected within the linear detection range. GAPDH or β‑tubulin was used as the internal reference protein for both PAM and Marc‑145 samples. The relative expression of target proteins was normalized to the corresponding reference protein band intensity.

### Quantitative real-time polymerase chain reaction (qRT-PCR)

Total cellular RNA was extracted using an RNA rapid extraction kit (Fastagen, 220,011). RT-qPCR was performed on a CFX96 Real-Time PCR System (Bio-Rad) using the Evo M-MLV RT Kit (Accurate Biology) and SYBR qPCR Master Mix (Vazyme, Q711). Total RNA was extracted from samples and reverse‑transcribed into complementary DNA (cDNA). Absolute quantification of PRRSV RNA copies was performed by SYBR‑Green RT‑qPCR. A standard curve was established with serially diluted recombinant plasmids containing *PRRSV N* gene fragment, from which viral copy numbers were calculated. All qPCR reactions were performed in technical triplicate. Primer sequences are listed in Table 1. Relative gene expression was calculated using the 2^−ΔΔ*Ct*^ method, with *GAPDH* as the internal reference gene.

**Table 1:** **Primer sequences used for RT-qPCR**

Target	Primer	Sequence (5′-3′)
*mGAPDH*	Forward	TGATGACATCAAGAAGGTGGTGAAG
*mGAPDH*	Reverse	TCCTTGGAGGCCATGTGGGCCAT
*sGAPDH*	Forward	CCTTCCGTGTCCCTACTGCCAAC
*sGAPDH*	Reverse	GACGCCTGCTTCACCACCTTCT
*PRRSV nsp9*	Forward	CGGCGGCTTAGTTGTTACTG
*PRRSV nsp9*	Reverse	TCAACCTCACTGGCCACTTT
*sIL-6*	Forward	AATGCTCTTCACCTCTCC
*sIL-6*	Reverse	TCACACTTCTCATACTTCTCA
*sIL-10*	Forward	GTGAAGAGTGCCTTTAGC
*sIL-10*	Reverse	TCTATGTAGTTGATGAAGATGTC
*mIL-1β*	Forward	ACCTATCTTCCTCCACACAAGC
*mIL-1β*	Reverse	TCTGCTTGAGAGGTGCTGATG
*mIL-6*	Forward	GGTGTTGCCTGCTGCCTTCC
*mIL-6*	Reverse	TGAGATGCCGTCGAGGATGTACC
*mIL-8*	Forward	AGCTGGCGGTGGCTCTCTTG
*mIL-8*	Reverse	TGGGGTGGATAGGTTTGGAGTACG
*mIL-18*	Forward	GCTTGAATCTAAATTATCAGTC
*mIL-18*	Reverse	GAAGATTCAAATTGCATCTTAT
*PEDV N*	Forward	CAAGAACAGAAACCAGTCAAATGACC
*PEDV N*	Reverse	AGAGTGGAGGAGAATTCCCAAGG
*SADS-CoV N*	Forward	CTGACTGTTGTTGAGGTTAC
*SADS-CoV N*	Reverse	TCTGCCAAAGCTTGTTTAAC

m, monkey; s, swine

### Inhibition of virus entry assay

Marc‑145 cells were seeded in 12‑well plates at 8 × 10^5^ cells per well and precultured for 12 h before treatment. To characterize the stage‑dependent antiviral activity of HCQ against PRRSV, seven treatment groups (G1–G7) covering viral adsorption, internalization, intracellular replication, and progeny virion release were set up over a 24 h infection cycle (Figure 5A).

G1 (Mock control): cells were cultured in maintenance medium without PRRSV or HCQ for 24 h. G2 (PRRSV infection control): cells were inoculated with PRRSV at 4 °C for 2 h to allow viral adsorption. Unbound virions were removed by washing, and cells were continuously cultured in drug‑free medium at 37 °C until 24 h post-infection (hpi). G3 (full-cycle HCQ treatment): PRRSV was incubated with cells in the presence of 50 μM HCQ during the 4 °C adsorption phase (0–2 h). After washing, cells were maintained in medium containing 50 μM HCQ for the remaining infection period (2–24 h). G4 (HCQ restricted to the adsorption phase): cells were coincubated with PRRSV and 50 μM HCQ at 4 °C for 2 h. Unbound virus and residual HCQ were thoroughly washed away, and cells were cultured in drug-free medium until 24 hpi. This regimen assessed HCQ’s effect on PRRSV adsorption. G5 (HCQ restricted to the early entry phase): following virus adsorption without HCQ (0–2 h, 4 °C), cells were treated with 50 μM HCQ during the early internalization stage (2–5 h, 37 °C). After HCQ was removed, cells were cultured in drug-free medium until 24 hpi. G6 (HCQ restricted to the early replication phase): after complete viral adsorption and internalization (0–5 h), cells were exposed to 50 μM HCQ during the early viral replication stage (5–12 h). HCQ was then removed, and cells were cultured without treatment until 24 hpi. G7 (HCQ restricted to late replication and virion release): after virus adsorption and untreated early infection (0–12 h), cells were treated with 50 μM HCQ during the late replication and progeny release stage (12–24 h).

For the standalone attachment assay (mimicking the G4 regimen), cells were coincubated with PRRSV (MOI = 1) and 50 μM HCQ at 4 °C for 2 h to permit only viral binding. After three PBS washes, cells were cultured in drug-free medium for 24 h. Intracellular viral *nsp9* messemger RNA (mRNA) levels were quantified using RT-qPCR. For the standalone post‑entry replication assay (mimicking the G6 regimen), cells were inoculated with PRRSV at 4 °C for 2 h, followed by incubation at 37 °C for 3 h to complete viral internalization. Cells were then treated with 50 μM HCQ from 5 to 12 hpi. Culture supernatants were harvested at 24 hpi for quantification of viral genomic copies via RT‑qPCR.

### Confocal immunofluorescence assay

Briefly, Marc-145 cells were transfected with the indicated plasmids for 24 h and fixed in 4% paraformaldehyde for 30 min. After three washes with PBS, the cells were treated with 0.1% Triton X-100 for 30 min at room temperature. After 1-h incubation with 5% bovine serum albumin (BSA), the cells were incubated with the appropriate primary antibodies overnight at 4 °C, followed by a 1-h incubation at room temperature with fluorescently labeled secondary antibodies. Finally, the cell nuclei were counterstained with 4′,6-diamidino-2-phenylindole for 5 min and fluorescence images were acquired using a laser scanning confocal immunofluorescence microscope (Olympus; Tokyo, Japan). Signal overlaps between different channels were analyzed using FluoView FV10i software (Olympus FV10i; Japan). Quantification of mCherry‑EGFP‑LC3 puncta was performed using Fiji‑ImageJ software. Over 10‑15 LC3‑reporter‑positive cells were randomly selected for each group across three independent biological replicates. Total EGFP-positive and total mCherry-positive puncta were quantified separately. EGFP and mCherry single‑channel raw images were used instead of merged composite images. Statistical analysis was performed by one‑way analysis of variance (ANOVA) followed by Tukey’s multiple‑comparisons post‑hoc test.

### In vivo PRRSV infection and treatment

All animal experiments complied with national laboratory animal welfare standards and the Animal Research: Reporting of In Vivo Experiments (ARRIVE) guidelines. The animal experimental protocol was approved by the Animal Management and Ethics Committee of South China Agricultural University (approval no. 2024c091).

A total of eighteen 28-day-old crossbred piglets (Duroc × Landrace × Yorkshire) with consistent body weight and healthy physical status were selected for this experiment. Piglets were randomly assigned into three experimental groups using a complete random grouping method (*n* = 6 per group): blank control group, PRRSV single-challenge group, and HCQ-treated PRRSV challenge group. The random grouping strategy was adopted to eliminate differences in individual piglet physique, immunity and baseline health status, thereby reducing experimental bias. Piglets in the blank control group received only intratracheal injection of equal-volume culture medium without viral inoculation. Piglets in the two challenge groups were intratracheally challenged with the field PRRSV strain XH-GD at a uniform dose of 2 × 10^5^ TCID_50_ per piglet; this inoculation dosage was determined based on preliminary preexperiment results to establish stable PRRSV infection with typical clinical and pathological lesions without rapid lethal shock. Piglets in the HCQ + PRRSV group received daily intramuscular HCQ administration at a dose of 6.5 mg/kg body weight throughout the whole 14-day experimental period starting on the day of viral challenge.

After viral inoculation, rectal temperature, respiratory clinical signs (cough, dyspnea), feed intake, and survival status of all piglets were monitored and recorded daily. Body weight was measured daily to evaluate growth performance, and peripheral blood samples were collected at corresponding time points for subsequent viral load and cytokine quantification. At 14 days post-infection (14 dpi), all surviving piglets were humanely euthanized for post-mortem examination. Piglets were anesthetized by intramuscular injection of xylazine‑ketamine mixture, and then humanely euthanized via carotid artery exsanguination. All animal experiments were performed in accordance with local animal welfare requirements and the ARRIVE guidelines. Lung tissues were harvested for histopathological scoring.

### Pathological examination

To evaluate the protective effects of HCQ against PRRSV infection, pathological changes in the lungs of all piglets were examined post-mortem at 14 dpi. Lung tissue samples were collected from each piglet, fixed in 10% neutral-buffered formalin, embedded in paraffin, cut into sections, and prepared for histological analysis. Thereafter, the sections were stained with hematoxylin and eosin (H&E) and examined for pathological changes using a microscope.

### Enzyme-linked immunosorbent assay (ELISA)

PRRSV ELISA antibody detection kit (PRRSV.001) was purchased from Qingdao Real Bio‑Technology Company. Pig interferon (IFN)‑β (CSB‑E13214P), IFN‑γ (CSB‑E06780P), IL‑6 (CSB‑E06786P), and IL‑10 (CSB‑E06779P) kits were purchased from CUSABIO. Briefly, serum samples were added to precoated microplates. After blocking with BSA to reduce nonspecific binding, plates were washed to remove unbound material, enzyme‑conjugated secondary antibody was added to bind target analytes. Plates were washed, substrate was added for color development, stop solution terminated reaction, and absorbance values were measured using an ELISA reader.

### Detection of viral load in serum

Viral RNA was extracted from 200 μL serum samples using a viral RNA extraction kit (Fastagen, 220011). The PRRSV RNA copy number was determined by RT-qPCR using a standard curve constructed with a recombinant plasmid containing the *PRRSV N* gene fragment. RT-qPCR was performed on a CFX96 Real-Time System (Bio-Rad) with SYBR qPCR Master Mix (Vazyme, Q711). All tests were performed in triplicate to ensure reproducibility.

### Network pharmacology prediction and enrichment analysis

#### Target collection of HCQ and PRRSV

Potential protein targets of HCQ were retrieved from SwissTargetPrediction, PharmMapper and PubChem databases, and unified to official human gene symbols for subsequent analysis. Host proteins interacting with PRRSV were screened from GeneCards, DisGeNET, and UniProt databases with a relevance score ≥ 1 as the threshold. Duplicate targets were removed using Excel software, and overlapping targets shared by HCQ and PRRSV were visualized by Venn diagrams using TBtools.

#### Construction of protein–protein interaction (PPI) network

The overlapping HCQ-PRRSV targets were imported into the Search Tool for the Retrieval of Interacting Genes/Proteins (STRING) database (version 11.5) to construct a protein–protein interaction network, with the species set as *Homo sapiens* and a confidence score > 0.7 as the screening cutoff. The raw interaction data exported from STRING was imported into Cytoscape 3.9.1 software for visualization. Node layout was rearranged according to degree centrality values, and interleukin-6 (IL-6) and acute phase proteins (APP) were screened out as core hub genes based on top-ranked degree values.

#### GO and KEGG enrichment analysis

Gene Ontology (GO) enrichment covering biological process (BP), cellular component (CC), molecular function (MF), and Kyoto Encyclopedia of Genes and Genomes (KEGG) pathway enrichment were performed on overlapping targets using the clusterProfiler package in R software (v4.2.1). Terms with adjusted *p*-value < 0.05 were regarded as significantly enriched. The top 10 enriched entries of each GO category and the top ten enriched KEGG pathways were selected for graphical visualization according to −log_10_(adjusted *p*-value).

### Statistical analysis

All statistical analyses were performed using GraphPad Prism software (version 8.0). Data are presented as mean ± standard deviation (SD). For comparisons between two independent groups, the two-tailed Student’s *t*-test was applied to determine statistical differences. For comparisons among three or more experimental groups, one-way analysis of variance (ANOVA) followed by Tukey’s multiple-comparisons post hoc test was used. The statistical model used for each dataset is specified in the corresponding figure legend and results section.

## Results

### HCQ inhibited PRRSV replication in vitro

**Figure 1:**
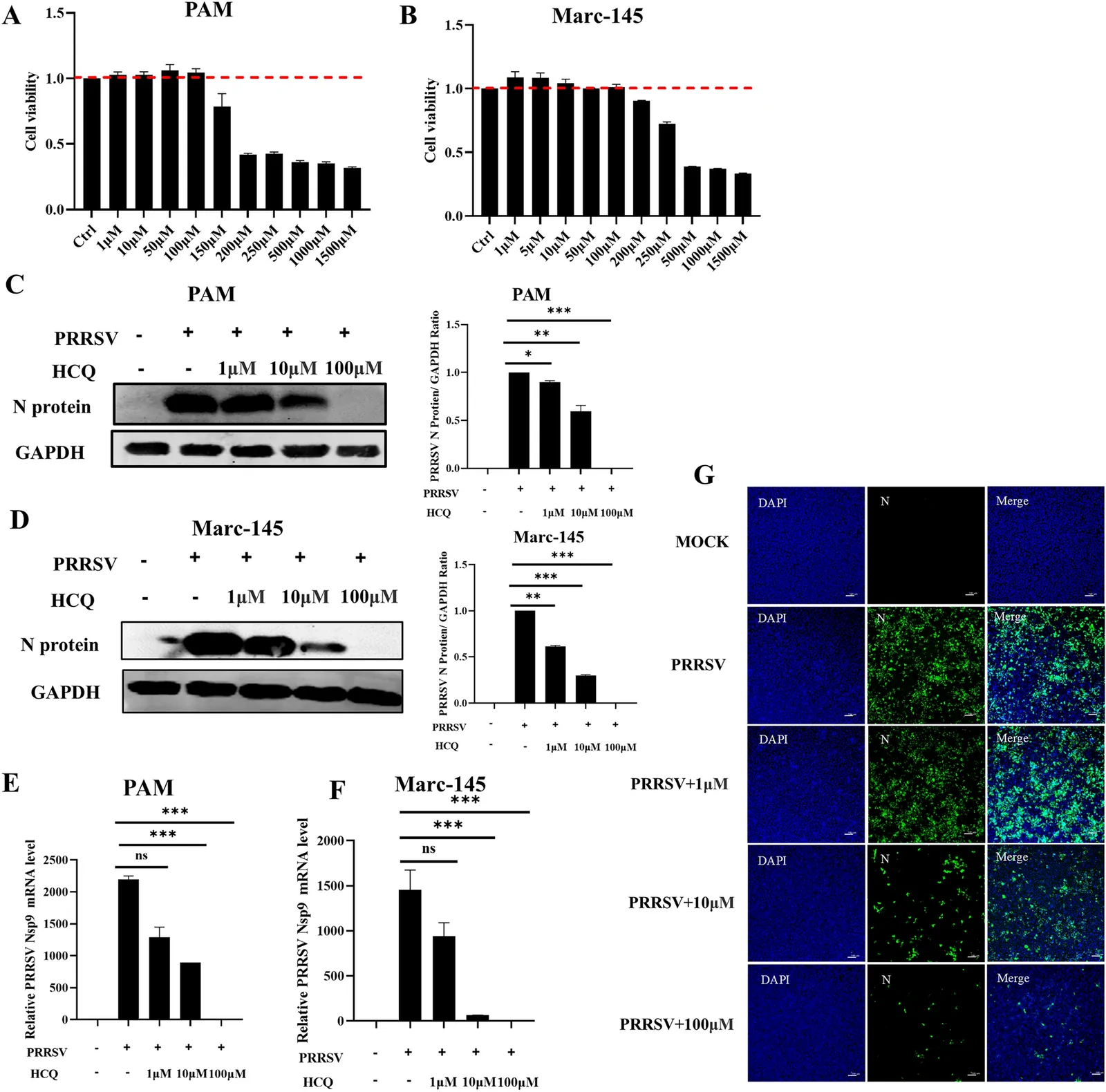
**HCQ inhibits PRRSV replication in vitro.** **A**, **B** PAMs and Marc-145 cells were pretreated with indicated doses of HCQ for 36 h, followed by cell viability assessment using the CCK-8 cell assay kit. Untreated cells served as the 100% control. **C** PAMs and **D** Marc‑145 cells infected with PRRSV (MOI = 1) for 24 h were pretreated with varying concentrations of HCQ (1, 10, and 100 μM) for 24 h, followed by western blot using anti‑PRRSV‑N antibodies. Gray‑scale quantification was performed for key proteins. **E** PRRSV RNA copy numbers in the supernatants of PAMs and **F** Marc‑145 treated cells were analyzed using qPCR. Note that **E** and **F** represent independent datasets from two distinct cell types; similar trends reflect consistent antiviral activity of HCQ, rather than duplicated data. Statistical significance was determined using one‑way ANOVA followed by Tukey’s multiple‑comparisons post hoc test; *p < 0.05, **p < 0.01, and ***p < 0.001. **G** Marc‑145 cells pretreated with HCQ (1, 10, and 100 μM) for 24 h were subjected to indirect immunofluorescence assay (IFA) using anti‑PRRSV‑N antibody. Scale bar, 100 μm. Data are presented as mean ± SD from three independent biological replicates, with three technical replicates per biological replicate.

CCK-8 assays showed negligible cytotoxicity at HCQ concentrations ≤ 100 μM in both cell types (Figure 1A, B). Western blotting and RT-qPCR demonstrated dose-dependent reductions in PRRSV N protein and viral RNA levels, with the strongest inhibition observed at 100 μM HCQ (Figure 1C–F). Consistently, indirect immunofluorescence showed reduced PRRSV infection intensity with increasing HCQ concentrations (Figure 1G). Together, these findings demonstrate that HCQ inhibits PRRSV replication in cultured cells within the tested noncytotoxic concentration range.

### HCQ significantly increased the survival rate of PRRSV-infected piglets

**Figure 2:**
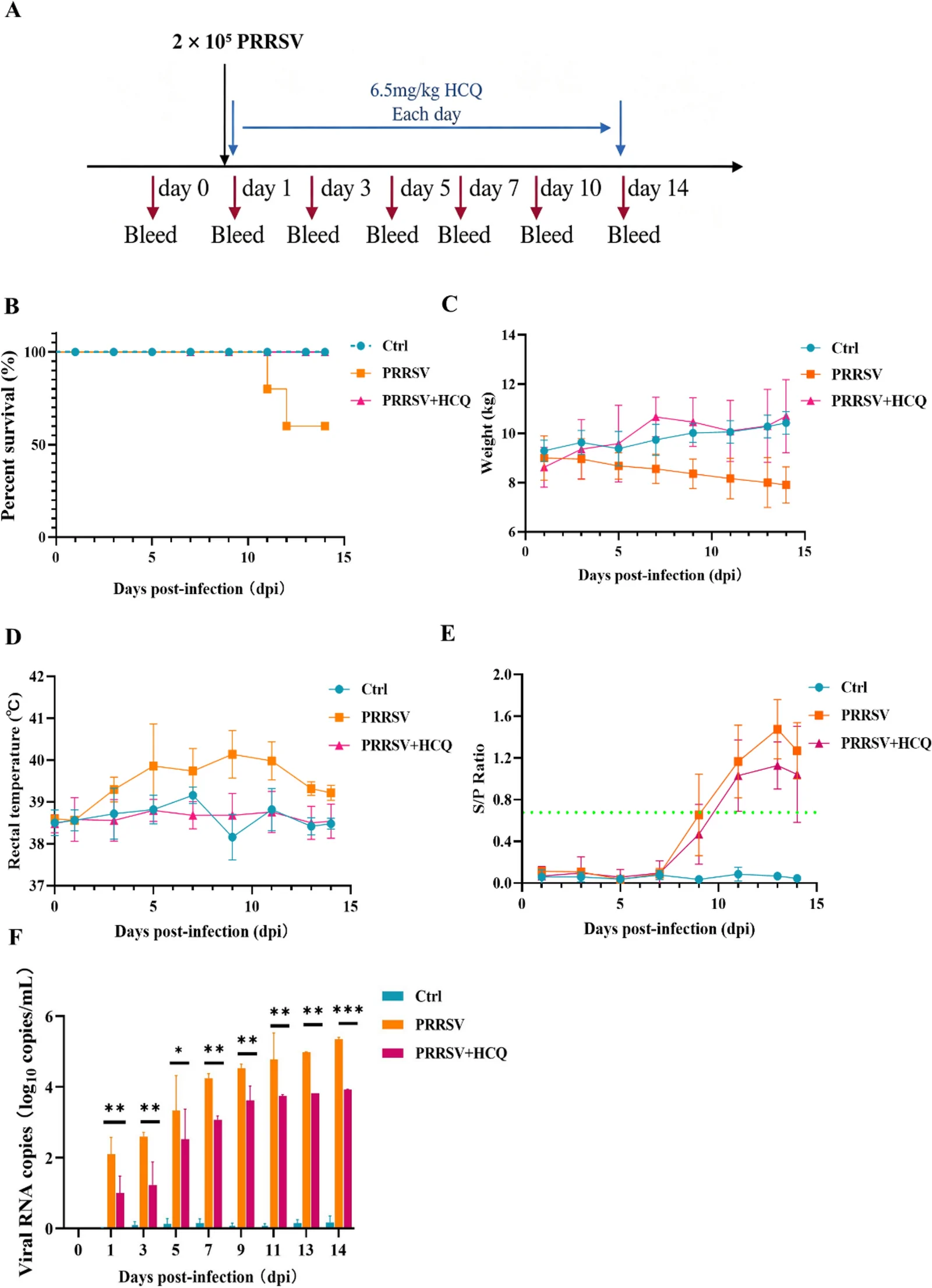
**HCQ treatment significantly increased the survival rate of piglets infected with PRRSV**. **A** Experimental design schematic. HCQ (6.5 mg/kg) was administered via intramuscular injection, and blood samples were collected at indicated time points. Piglets were euthanized at 14 days post-infection (dpi) to collect blood, lung tissue, and other pathological specimens. **B** Daily survival rates were monitored for piglets in the negative control, positive control, and HCQ treatment groups. **C** Daily body weight changes. **D** Body temperature changes. **E** Serum N-protein antibody levels in piglets at indicated time points were detected using an ELISA kit. **F** Viral load in serum from PRRSV-infected piglets. Data are presented as mean ± SD (*n* = 6 animals per group). Statistical significance was determined using one-way ANOVA followed by Tukey’s multiple-comparisons post hoc test; *p < 0.05, **p < 0.01, ***p < 0.001 and ****p < 0.0001.

Animal experiments were conducted to investigate the antiviral effects of HCQ against PRRSV in vivo. Eighteen piglets (4-weeks-old) were randomly divided into three groups: control, PRRSV, and HCQ + PRRSV (Figure 2A). Notably, intramuscular HCQ conferred robust protection against lethal PRRSV challenge in 4-week-old piglets. At 14 days post-infection (14 dpi), the PRRSV-only group displayed a mortality rate of 33.3% (two out of six piglets), while zero mortality was recorded in the HCQ-treated challenged group (Figure 2B). Additionally, piglets in the HCQ + PRRSV group showed a gradual increase in average body weight compared with those in the PRRSV group, maintaining a relatively stable growth trend consistent with that of the control group (Figure 2C). Moreover, no significant difference was observed in average body temperature between the HCQ + PRRSV and control groups (Figure 2D). In contrast, piglets in the positive control group (PRRSV) exhibited an elevated body temperature. ELISA and RT-qPCR revealed that HCQ treatment suppressed PRRSV replication in piglets (Figure 2E, F). Collectively, these results indicate that HCQ effectively ameliorated PRRSV-induced clinical symptoms in piglets, including weight loss and fever.

### HCQ inhibited pathological changes in lungs of piglets infected with PRRSV

**Figure 3:**
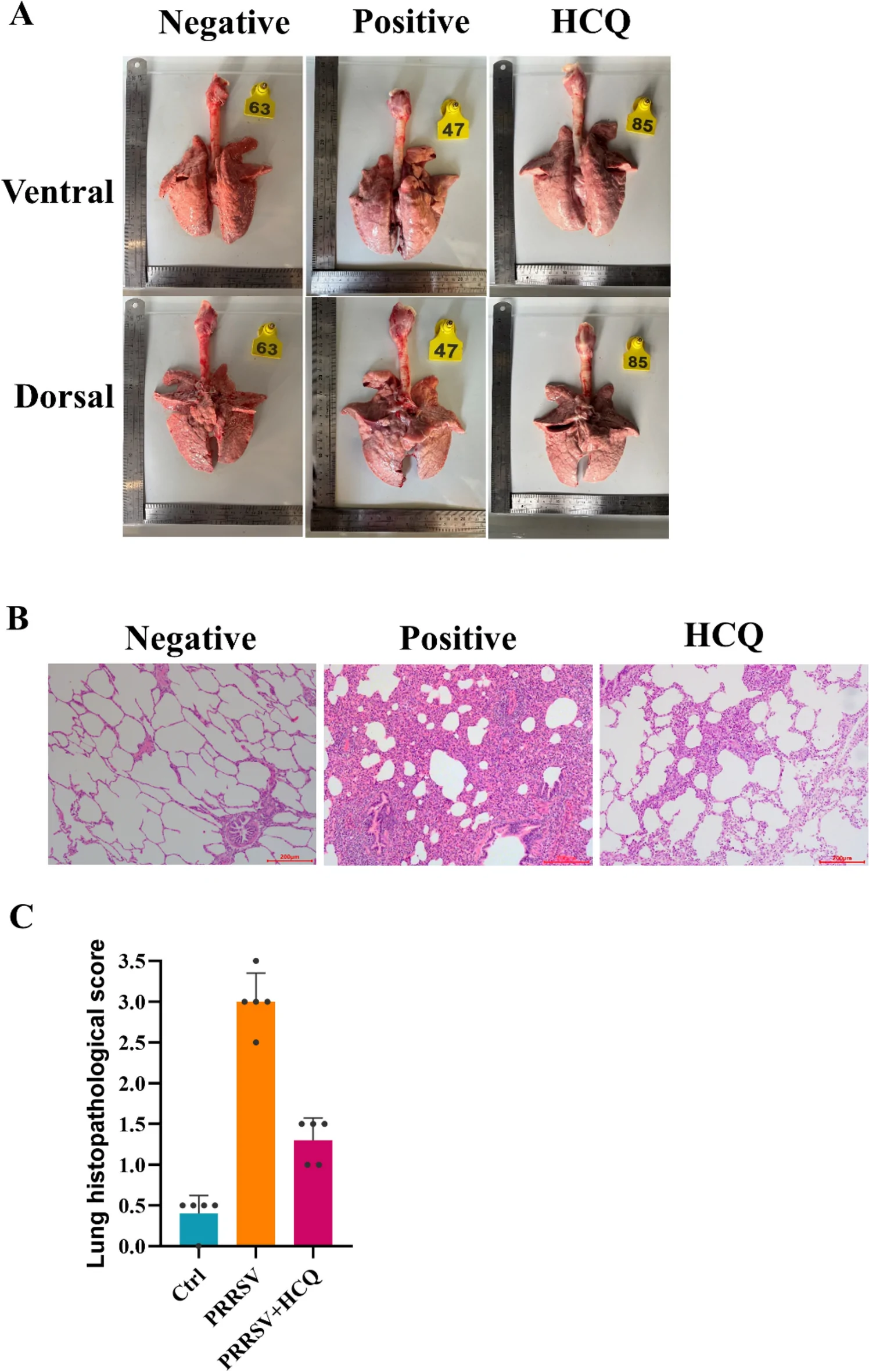
**HCQ inhibits pathological changes in lungs of piglets infected with PRRSV**. **A** Piglets were euthanized at 14 days post-infection, and gross histopathological lesions were examined in the lungs of piglets in the negative control, positive control, and HCQ treatment groups. **B** Representative H&E-stained lung sections in sections stained with hematoxylin and eosin in the negative control, positive control, and HCQ treatment groups. Scale bar, 200 μm. **C** Quantitative representation of histopathological damage scores in lung tissues across different groups. Data are presented as mean ± SD (*n* = 6 animals per group). Statistical significance was determined using one-way ANOVA followed by Tukey’s multiple-comparisons post hoc test.

Gross pathological examination revealed intact lung tissue without lesions in the blank control group. In contrast, piglets in the PRRSV group displayed diffuse pulmonary enlargement, congestion, tissue hardening, and thickened gray-white visceral pleura and pulmonary adhesions. The HCQ-treated group only presented mild gray-white pleural lesions without severe pulmonary congestion or consolidation (Figure 3A). Histopathological H&E staining showed severe interstitial pneumonia in PRRSV-infected piglets, characterized by thickened alveolar septa, massive interstitial inflammatory cell infiltration and destroyed lung tissue architecture. Only mild inflammatory cell infiltration was observed in lungs from HCQ-treated piglets (Figure 3B). Quantitative pathological scoring confirmed significantly higher lung lesion scores in the PRRSV group relative to both the blank control and HCQ + PRRSV groups (Figure 3C). Although we comprehensively evaluated gross lesions and histological inflammatory injury in lung tissues, viral RNA loads in lung parenchyma were not quantified in this experiment. The attenuated pulmonary pathological damage observed in HCQ-treated piglets could be partially explained by reduced systemic viremia detected in serum, yet direct evidence linking local lung viral burden to pathological relief is absent. These results indicate HCQ markedly alleviates PRRSV-triggered interstitial lung injury.

### Effects of HCQ on interferon and inflammation levels in PRRSV-infected pigs

**Figure 4:**
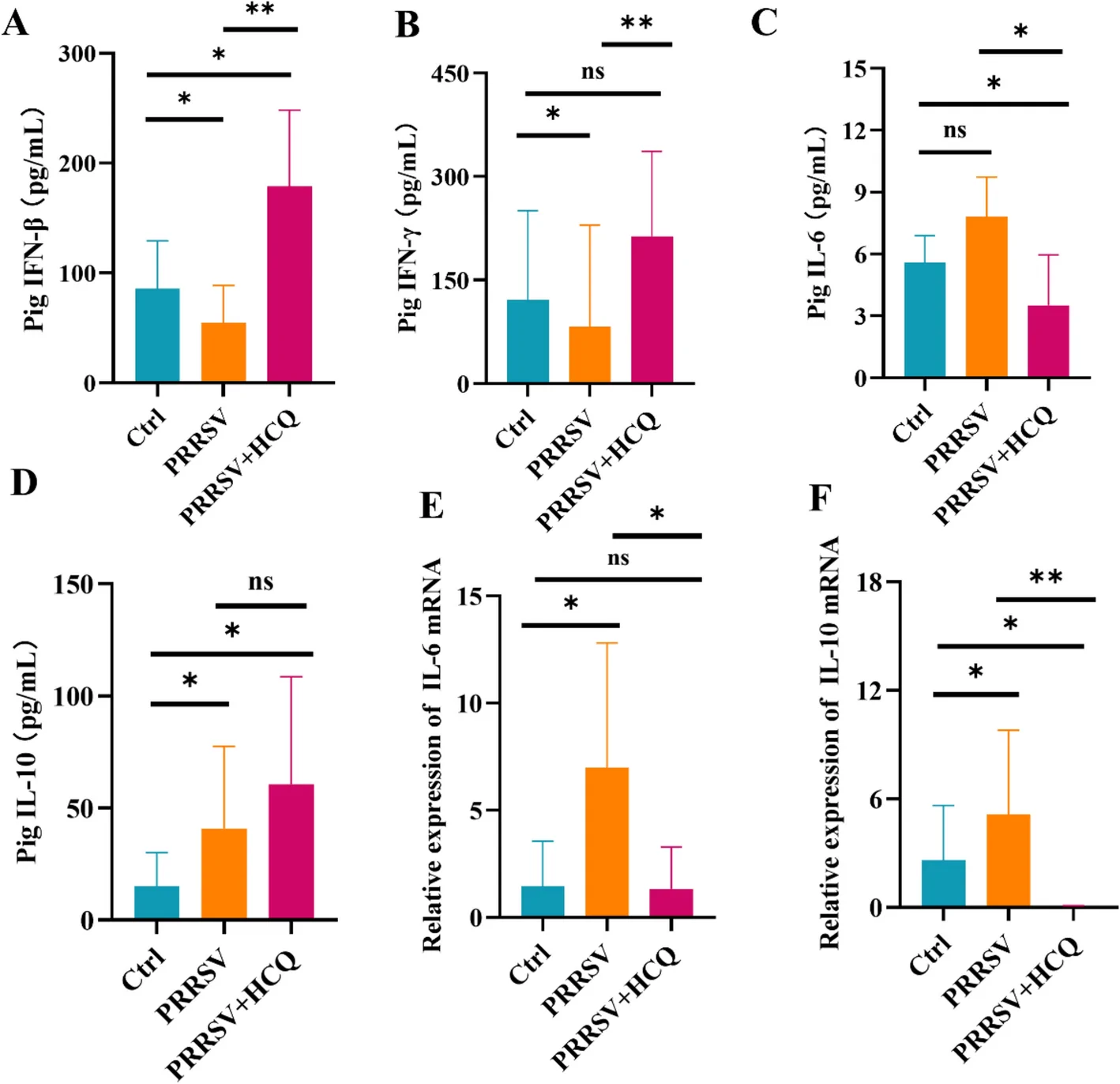
**Effects of HCQ on interferon and inflammation levels in piglets infected with PRRSV**. **A**, **B** After euthanizing piglets at 14 dpi, serum IFN-β and IFN-γ levels were detected in the negative control, positive control group, and HCQ treatment groups using an ELISA kit. **C**, **D** Serum levels of IL-6 and IL-10, were detected in the negative control, positive control, and HCQ-treated groups using an ELISA kit. **E**, **F** RT-qPCR was performed to detect IL-6 and IL-10 mRNA levels in the tissues of piglets in the negative control, positive control, and HCQ-treated groups. Statistical significance was determined using one-way ANOVA followed by Tukey’s post hoc test; *p < 0.05, **p < 0.01, ***p < 0.001, ****p < 0.0001. Data are presented as mean ± SD (*n* = 6 animals per group).

We first detected serum type I and II interferon levels to evaluate HCQ’s immunomodulatory effect during PRRSV infection. ELISA results showed that serum IFN-β and IFN-γ concentrations were markedly reduced in PRRSV-challenged piglets relative to uninfected controls, whereas HCQ treatment partially restored interferon production (Figure 4A, B). Given the important contribution of inflammatory dysregulation to PRRSV-associated pathology, we further focused on the expression of key inflammatory and immune-regulatory cytokines. ELISA assays revealed that PRRSV infection drastically increased the serum protein levels of proinflammatory IL-6 and immunosuppressive IL-10. HCQ intervention effectively suppressed excessive IL-6 secretion and partially reversed virus-mediated IL-10 elevation, demonstrating HCQ can alleviate PRRSV-triggered inflammatory injury and relieve virus-induced immune imbalance (Figure 4C, D). RT-qPCR analysis yielded consistent transcriptional trends. PRRSV infection upregulated IL-6 mRNA expression to nearly six times that of the control group (*p* < 0.05) and doubled IL-10 transcript abundance (*p* < 0.05). HCQ administration significantly downregulated both IL-6 and IL-10 transcription, returning their expression levels to values comparable to the blank group (Figure 4E, F). Although HCQ moderately rescued PRRSV-suppressed interferon synthesis, its dominant protective effects may be associated with restraint of overactive inflammatory signaling and subsequent alleviation of inflammatory injury and immune imbalance in piglets.

### Effects of HCQ on different stages of PRRSV infection

**Figure 5:**
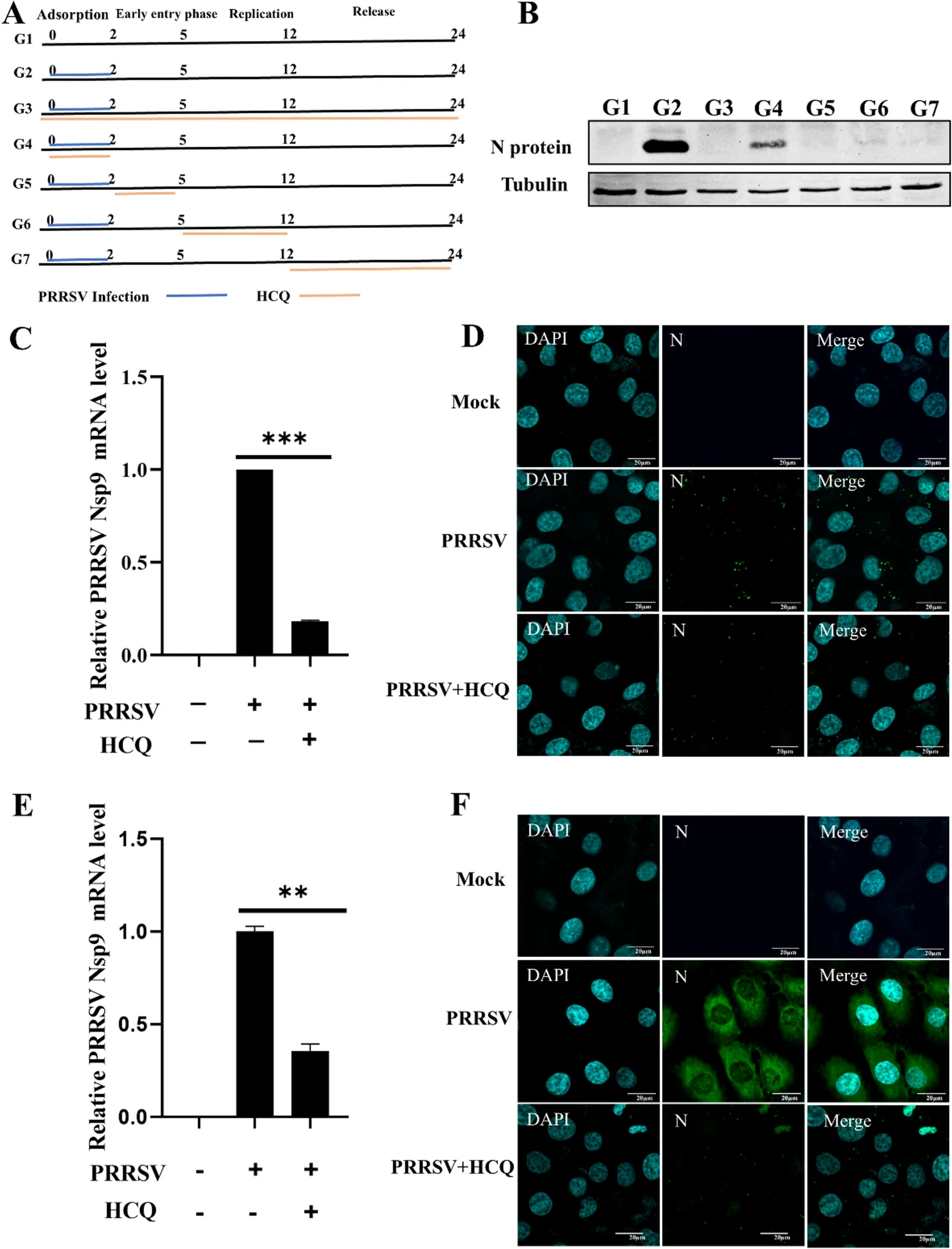
**Effects of HCQ on different stages of PRRSV infection**. **A** Schematic timeline of seven distinct treatment regimens (G1–G7) within a 24-h infection cycle, with full step-by-step operation details for each group described in Section Inhibition of virus entry assay. Blue horizontal bars represent the 0–2 h PRRSV inoculation period for viral adsorption; yellow horizontal bars denote the time window when 50 μM HCQ was supplemented to cell culture medium for each group. G4 exclusively targets viral adsorption (4 °C cotreatment with virus and HCQ); G5 early entry phase (HCQ added 2–5 h post viral adsorption); G6 targets early intracellular viral replication (HCQ added 5–12 h post complete virion entry). **B** Western blot analysis of PRRSV N protein expression in Marc-145 cells subjected to G1–G7 treatment regimens, with Tubulin as a loading control. **C**, **D** RT-qPCR quantification of PRRSV nsp9 mRNA and immunofluorescence detection of viral N protein in cells treated under the G4 adsorption-phase regimen. **E** RT‑qPCR detection of PRRSV nsp9 mRNA in cells treated under the G5 early post-attachment treatment‑window regimen. **F** Immunofluorescence staining of PRRSV N protein in cells treated under the G6 intracellular replication-phase regimen. Scale bar, 20 μm. Statistical significance was determined using one-way ANOVA followed by Tukey’s multiple-comparisons post hoc test; *p < 0.05, **p < 0.01, ***p < 0.001, ****p < 0.0001. Data are presented as mean ± SD from three independent biological replicates, with three technical replicates per biological replicate.

To investigate the stage-dependent antiviral activity of HCQ during the PRRSV life cycle, we established seven time-controlled treatment regimens (G1–G7) to evaluate the effects of HCQ intervention at different phases of infection, including viral adsorption, post-adsorption entry events, and intracellular replication (Figure 5A). Western blot analysis showed that HCQ treatment applied at different intervention windows consistently reduced PRRSV N protein expression compared with the untreated infection control group (G2) (Figure 5B), suggesting that HCQ exerts inhibitory effects throughout multiple stages of PRRSV infection.

We further evaluated the effects of HCQ during individual infection phases using stage-specific treatment regimens. Under the adsorption-phase regimen (G4), HCQ exposure during viral attachment significantly decreased PRRSV nsp9 mRNA levels and reduced viral N protein-positive signals compared with the PRRSV control group (Figure 5C, D), indicating that HCQ interferes with early virus–cell interaction events. These results indicate that HCQ exposure during the adsorption period reduces subsequent PRRSV infection efficiency, although direct effects on physical virion attachment require further validation. In addition, HCQ treatment within the early post-attachment treatment-window (G5) also resulted in reduced viral genome levels (Figure 5E), suggesting that HCQ may affect early events following viral attachment. However, because virion internalization and early intracellular processes partially overlap within this time window, further specialized assays are required to precisely distinguish the effect of HCQ on virion internalization from subsequent post-entry events.

To assess the effect of HCQ after viral entry, cells were treated with HCQ during the early post-entry intracellular phase (G6). Confocal immunofluorescence analysis demonstrated decreased PRRSV N protein accumulation following HCQ treatment (Figure 5F), indicating that HCQ retains antiviral activity during early intracellular infection. Collectively, these findings demonstrate that HCQ exerts multistage antiviral effects during PRRSV infection, affecting viral attachment-associated events and subsequent intracellular replication-associated processes, rather than acting on a single step of the viral life cycle.

### Network pharmacology analysis identifies potential targets and pathways associated with hydroxychloroquine against PRRSV

**Figure 6:**
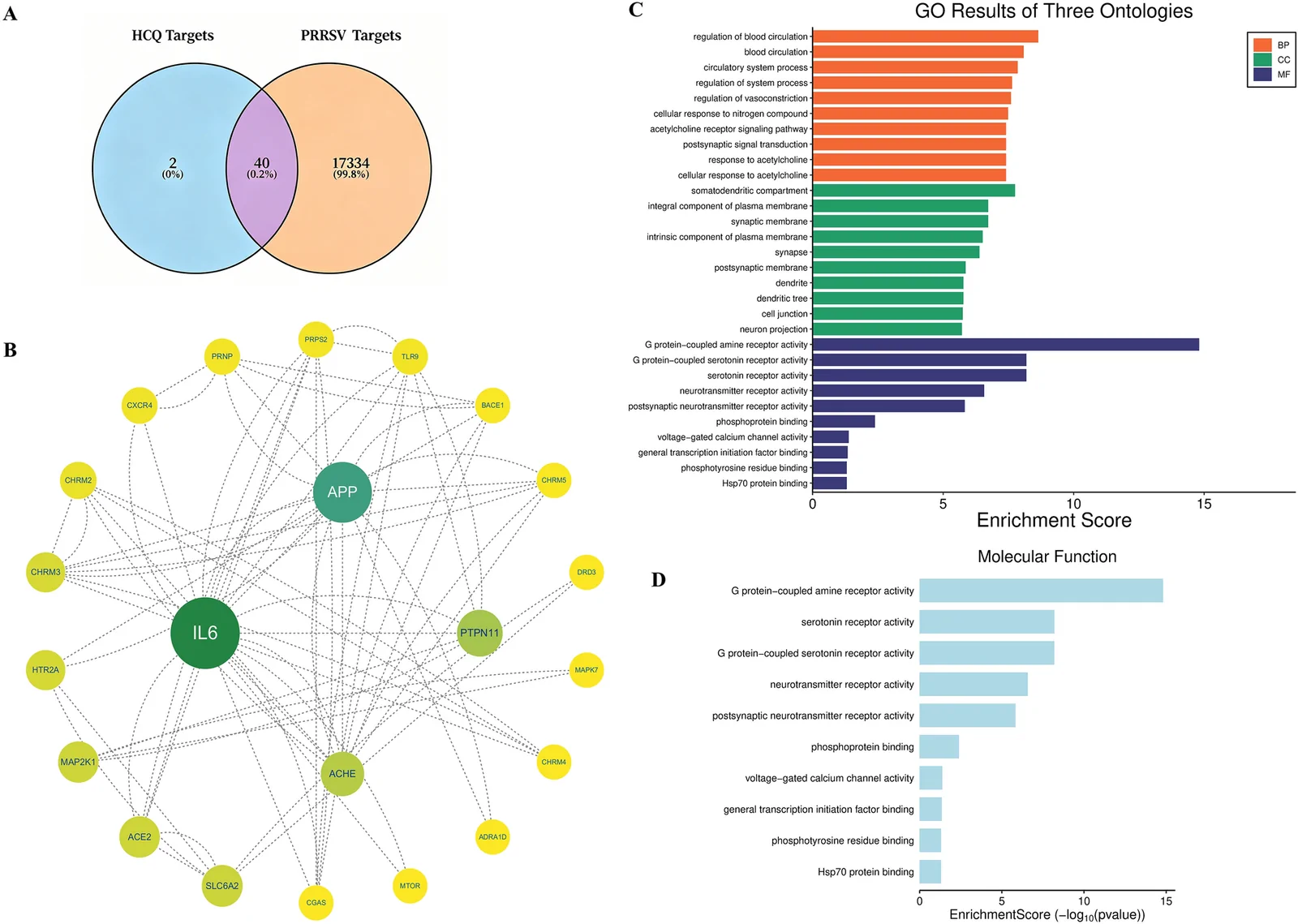
**Network pharmacology analysis identifies potential targets and pathways associated with hydroxychloroquine against PRRSV**. **A** Venn diagram of overlapping targets between HCQ and PRRSV. **B** PPI network constructed using the STRING database and Cytoscape software. **C** GO enrichment analysis of HCQ-PRRSV overlapping targets. The top ten enriched terms for biological processes (BP, orange), cellular components (CC, green), and molecular functions (MF, blue) are displayed according to enrichment score [−log_10_(adjusted *p*-value)]. **D** KEGG pathway enrichment analysis of HCQ-PRRSV overlapping targets. The top ten enriched pathways are ranked according to enrichment score [−log_10_(adjusted *p*-value)].

To explore potential molecular targets and biological processes associated with the antiviral effects of HCQ against PRRSV, network pharmacology analysis was performed. A total of 42 potential HCQ-associated targets and 17,374 PRRSV-related host targets were collected from public databases, resulting in 40 overlapping candidate targets between HCQ and PRRSV (Figure 6A). Protein–protein interaction (PPI) network analysis further identified highly connected candidate proteins, among which IL-6 and APP exhibited relatively high degree values and were selected as predicted hub genes (Figure 6B). These findings suggest that IL-6 and APP may represent potential regulatory nodes associated with the biological effects of HCQ during PRRSV infection. However, the functional involvement of APP in HCQ-mediated antiviral activity requires further experimental validation.

GO enrichment analysis revealed that the overlapping targets were mainly associated with biological processes related to circulatory regulation, cellular signaling, and protein regulation. Cellular component analysis indicated enrichment in membrane-associated and vesicle-related structures, whereas molecular function analysis highlighted receptor-associated and regulatory activities (Figure 6C). These enriched terms suggest that HCQ-associated targets may participate in multiple cellular processes involved in host responses to PRRSV infection.

KEGG pathway enrichment analysis identified several significantly enriched pathways, including neuroactive ligand–receptor interaction, cholinergic synapse, and calcium signaling pathways (Figure 6D). These pathways may reflect potential biological processes linking HCQ-associated targets with host inflammatory regulation and cellular homeostasis during viral infection. Collectively, network pharmacology analysis provides a theoretical framework for identifying candidate targets and pathways associated with HCQ-mediated protection against PRRSV, which were further investigated through subsequent cellular validation experiments.

### HCQ suppresses PRRSV-triggered NF-κB inflammatory signaling

**Figure 7:**
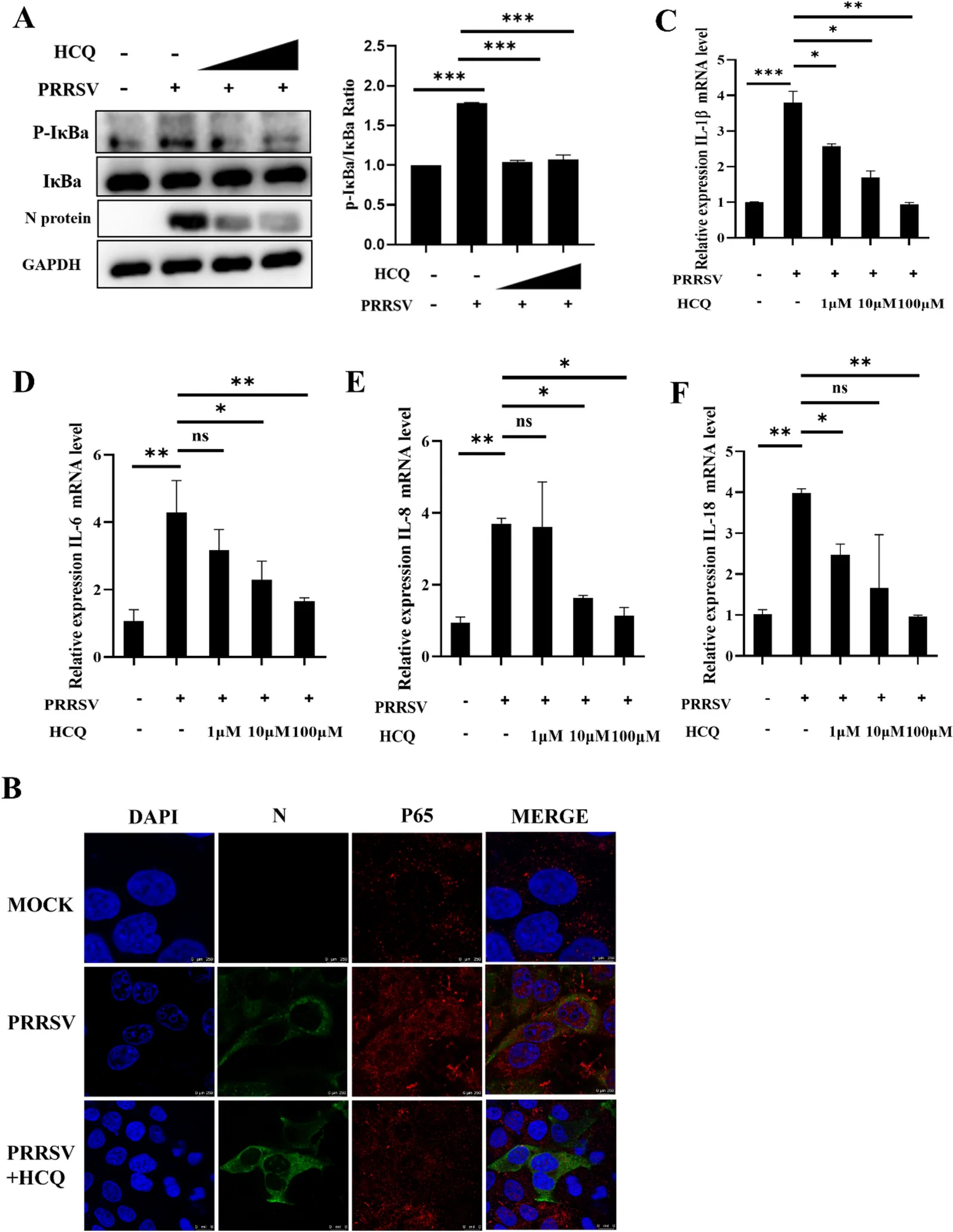
**HCQ suppresses PRRSV-triggered NF-κB inflammatory signaling**. **A** Western blot analysis of p-IκBα, total IκBα, PRRSV N protein, and GAPDH in Marc-145 cells. Cells were pretreated with increasing concentrations of HCQ (0, 20, 50 μM) for 2 h, followed by PRRSV infection (MOI = 1) for 24 h. GAPDH was used as a loading control. Quantitative analysis of the p-IκBα/IκBα ratio. Statistical significance for the quantitative analysis of the p-IκBα/IκBα ratio was determined using one-way ANOVA followed by Tukey’s multiple-comparisons post hoc test. **B** Confocal immunofluorescence analysis of Marc-145 cells infected with PRRSV (MOI=1) for 24 h, with or without HCQ pretreatment. Scale bar, 100 μm. **C**–**F** Marc-145 cells were infected with PRRSV at an MOI of 1 and treated with 1, 10, or 100 μM HCQ for 24 h. The mRNA levels of IL-1β, IL-6, IL-8, and IL-18 were detected by RT-qPCR. All quantitative data are presented as mean ± SD. Each group was compared with the PRRSV-infected group. Statistical significance was determined using one-way ANOVA followed by Tukey’s multiple-comparisons post hoc test; ^∗^*p* < 0.05, ^∗∗^*p* < 0.01, ^∗∗∗^*p* < 0.001, and ^∗∗∗∗^*p* < 0.0001. Data are presented as mean ± SD from three independent biological replicates, with three technical replicates per biological replicate.

Western blot results showed that PRRSV infection significantly induced the phosphorylation (p‑IκBα) and degradation of IκBα, and HCQ treatment markedly attenuated this process in a dose‑dependent manner: the p‑IκBα/IκBα ratio decreased with increasing HCQ concentration, accompanied by a notable reduction in PRRSV N protein expression. These findings indicate that HCQ suppresses early activation of the PRRSV‑triggered NF‑κB signaling pathway and was associated with reduced phosphorylation and degradation of IκBα (Figure 7A). Confocal immunofluorescence revealed obvious nuclear translocation of NF‑κB p65 after PRRSV infection; HCQ treatment markedly retained p65 in the cytoplasm and reduced nuclear p65 accumulation (Figure 7B). RT‑qPCR further confirmed that PRRSV infection significantly upregulated mRNA levels of proinflammatory cytokines IL‑1β, IL‑6, IL‑8, and IL‑18, while HCQ downregulated these transcripts in a concentration‑dependent fashion (Figure 7C–F). These data, together with autophagy‑related phenotypes, reveal that HCQ treatment was associated with alterations in both PRRSV‑triggered autophagic flux and NF‑κB‑driven inflammatory cascades.

### HCQ disrupts PRRSV-induced autophagic flux

The mCherry‑EGFP‑LC3 reporter exploits distinct pH sensitivity of the two fluorophores. Yellow mCherry^+^EGFP^+^ puncta correspond to autophagosomes with neutral luminal pH before lysosome fusion. Red‑only mCherry^+^EGFP^−^ puncta mark autophagic structures exposed to acidic lysosomal lumen, as EGFP fluorescence is quenched under acidic conditions while mCherry remains stable.

**Figure 8:**
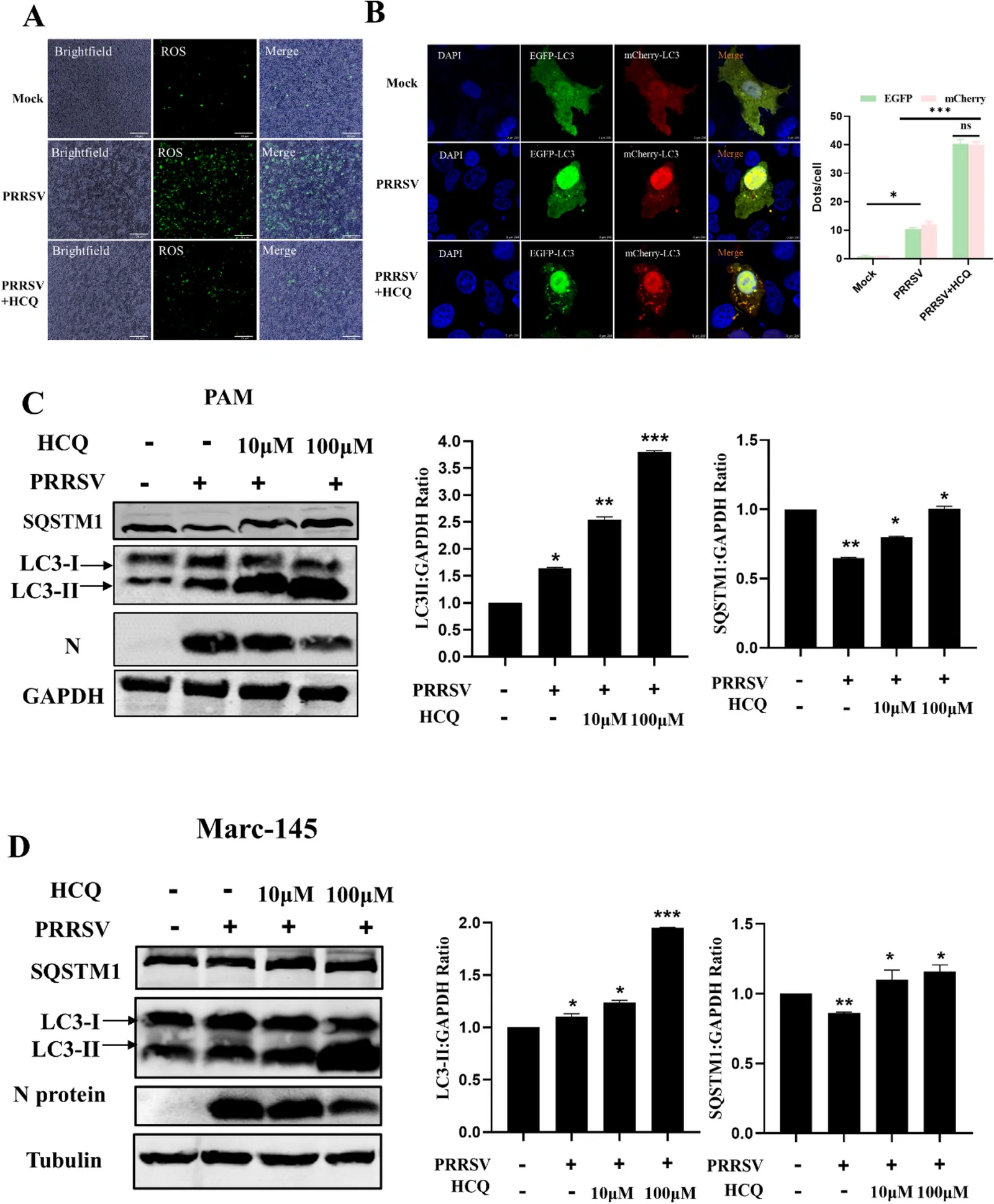
**HCQ disrupts PRRSV-induced autophagic flux**. **A** Marc‑145 cells were infected with PRRSV (MOI = 1) for 24 h. Intracellular ROS levels were measured after HCQ treatment using DCFH‑DA staining. Scale bar, 20 μm. **B** Marc‑145 cells were transfected with mCherry‑EGFP‑LC3 reporter. Total EGFP‑positive puncta and total mCherry‑positive puncta were manually counted from separate single‑channel raw images (not merged composite images). More than 10–15 LC3‑reporter‑positive cells were randomly selected for each group across three independent biological replicates. Scale bar, 100 μm. **C**, **D** PAM and Marc‑145 cells were infected with PRRSV (MOI = 1) for 24 h with or without HCQ treatment. Western blotting was performed to detect N protein, LC3‑I/‑II, and SQSTM1/p62. Grayscale quantification was performed for key protein bands. Statistical significance was determined using one‑way ANOVA followed by Tukey’s multiple‑comparisons post‑hoc test; *p < 0.05, **p < 0.01, ***p < 0.001, ****p < 0.0001. Data are presented as mean ± SD from eight independent biological replicates.

Quantitative puncta analysis of confocal single‑channel images revealed that PRRSV infection significantly increased the total numbers of EGFP‑positive and mCherry‑positive LC3‑reporter puncta per cell. These findings collectively suggest increased accumulation of LC3‑positive autophagic structures upon PRRSV infection. Notably, treatment with HCQ in PRRSV‑infected cells further elevated the abundance of reporter‑positive puncta (Figure 8B). Supporting this interpretation, HCQ treatment induced robust accumulation of LC3‑II and restored SQSTM1/p62 protein levels in PAM and Marc‑145 cells (Figure 8C, D). These observations are consistent with blockade of autophagic degradation. Meanwhile, HCQ‑mediated inhibition of autophagic turnover was accompanied by a significant reduction in intracellular reactive oxygen species (ROS) accumulation, suggesting that disrupted autophagy may be associated with altered redox homeostasis during PRRSV infection (Figure 8A). Collectively, these results indicate HCQ alters PRRSV‑triggered autophagic responses, accompanied by reduced intracellular ROS accumulation.

### Broad-spectrum in vitro antiviral activity of HCQ against diverse PRRSV strains, PEDV, and SADS-CoV

**Figure 9:**
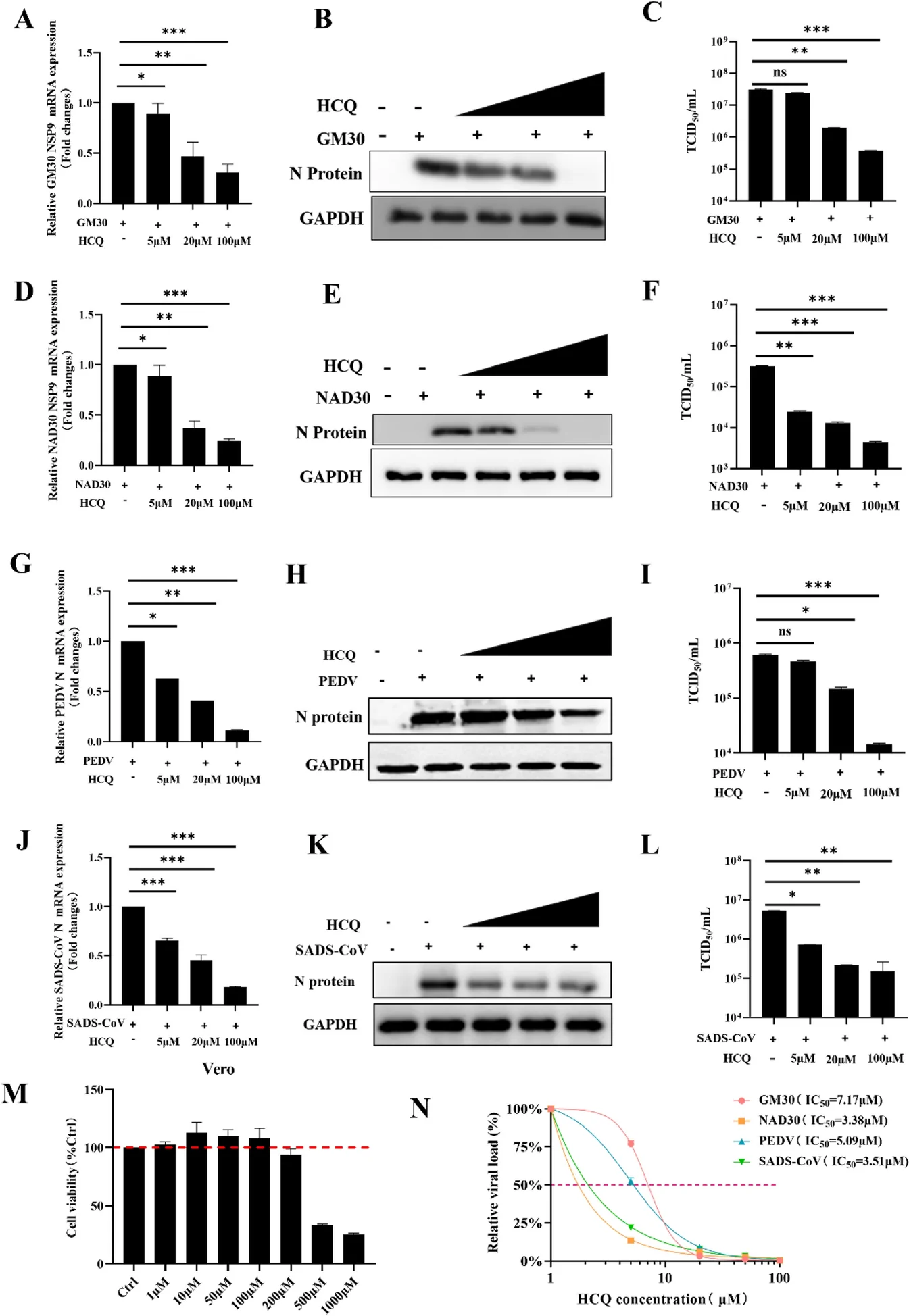
**Broad-spectrum in vitro antiviral activity of HCQ against diverse PRRSV strains, PEDV, and SADS-CoV**. **A**, **D** Relative mRNA expression of PRRSV ORF7 (strains GM30 and NAD30) in Marc-145 cells, as determined by RT-qPCR. GAPDH was used as the internal control. **B**, **E** Western blot analysis of PRRSV N protein in Marc-145 cells infected with PRRSV (GM30 or NAD30) and treated with graded concentrations of HCQ. GAPDH served as the loading control. **C**, **F** Viral titers in cell culture supernatants were measured by TCID_50_ assay. **G** Relative mRNA expression of PEDV N in Vero cells, analyzed by RT-qPCR. GAPDH was used as the internal control. **H** Western blot analysis of PEDV N protein in Vero cells infected with PEDV and treated with graded HCQ concentrations. GAPDH served as the loading control. **I** PEDV titers in cell culture supernatants were determined by TCID_50_ assay. **J** Relative mRNA expression of SADS-CoV N in Vero cells, analyzed by RT-qPCR. GAPDH was used as the internal control. **K** Western blot analysis of SADS-CoV N protein in Vero cells infected with SADS-CoV and treated with graded HCQ concentrations. GAPDH served as the loading control. **L** SADS-CoV titers in cell culture supernatants were measured by TCID_50_ assay. **M** Cell viability of Vero cells treated with the indicated concentrations of HCQ for 36 h, as determined by CCK-8 assay. **N** Dose–response curves and IC_50_ of HCQ against four swine RNA viruses. Data are presented as mean ± SD from three independent biological replicates, with three technical replicates per biological replicate. Statistical significance was determined using one-way ANOVA followed by Tukey’s multiple-comparisons post hoc test; *p < 0.05, **p < 0.01, ***p < 0.001, and ****p < 0.0001; ns, not significant.

Given that diverse swine RNA viruses hijack host autophagic machinery for replication, we further evaluated whether HCQ exerts broad-spectrum antiviral activity beyond PRRSV. RT-qPCR, western blotting, and TCID_50_assays showed that HCQ suppressed ORF7 transcription, N protein expression, and infectious viral titers of genetically divergent PRRSV field isolates, including NAD30, in a dose-dependent manner (Figure 9A–F). We next evaluated the antiviral activity of HCQ against PEDV and SADS-CoV in Vero cells and observed similar dose-dependent reductions in viral RNA levels, structural protein expression, and infectious viral titers (Figure 9G–L). HCQ showed no obvious cytotoxicity at concentrations up to 100 μM in Vero cells (Figure 9M). The IC_50_ values were 7.17 μM for PRRSV GM30, 3.38 μM for PRRSV NAD30, 5.09 μM for PEDV, and 3.51 μM for SADS-CoV, whereas the CC_50_ of HCQ exceeded 100 μM in both cell lines (Figure 9N). Accordingly, the selectivity index (SI), calculated as CC_50_/IC_50_, was > 14 for all tested viruses, with the highest selectivity observed for PRRSV NAD30. These findings indicate that HCQ exhibits broad-spectrum in vitro antiviral activity against diverse swine RNA viruses, including PRRSV, PEDV, and SADS-CoV, with a favorable selectivity profile in the tested cell models.

## Discussion

In this study, we demonstrated that HCQ protects against PRRSV infection in vitro and in vivo. In cultured cells, HCQ exerts obvious inhibitory effects at the viral attachment and post-entry replication stages of PRRSV life cycle and suppresses PRRSV-induced autophagic flux and NF-κB signaling. Conventional time-of-addition experimental design cannot fully separate virion internalization from subsequent post-entry intracellular events, so our current data cannot firmly confirm that HCQ directly blocks viral internalization. Further specialized independent assays are required to precisely clarify whether HCQ affects PRRSV endocytic uptake in follow-up research. In the piglet challenge model, HCQ reduced serum-derived systemic viremia, alleviated pulmonary histopathological lesions and overall disease severity in piglets. HCQ also exhibited broad-spectrum in vitro antiviral activity against divergent PRRSV strains, PEDV, and SADS-CoV in cell culture. However, the antiviral efficacy against PEDV and SADS-CoV requires further validation in animal challenge models. Although these viruses differ substantially in their viral families and replication strategies, accumulating evidence suggests that diverse swine RNA viruses exploit conserved host autophagy and endolysosomal pathways to facilitate viral entry, intracellular trafficking, replication, and/or maturation. This shared dependence on host vesicular and lysosomal machinery may provide a common vulnerability to lysosomotropic agents such as HCQ.

HCQ differs from chloroquine (CQ) by an aliphatic hydroxyl side chain, which contributes to its distinct pharmacokinetic and safety profiles. Although both agents inhibit autophagy by disrupting lysosomal acidification [26], HCQ generally exhibits a more favorable tolerability profile than CQ [27]. This combination of antiviral activity and improved safety supports HCQ as a potential repurposed candidate for PRRSV control [28].PRRSV can remodel autophagic processes to facilitate viral replication and disrupt immune homeostasis by targeting autophagy-related factors and endolysosomal pathways [29]. As a lysosomotropic agent, HCQ accumulates in lysosomes and neutralizes their acidic environment [30], thereby impairing lysosomal degradation and autophagosome–lysosome fusion [31]. Consistent with this mechanism, our findings show that HCQ inhibits PRRSV-induced autophagic flux and restricts viral replication. Given that autophagy and endolysosomal processes are commonly exploited by diverse RNA viruses, disruption of these conserved host pathways may contribute to the antiviral activity of HCQ observed against PEDV and SADS-CoV in vitro. Whether these mechanisms can confer protection in vivo remains to be determined.

Earlier preliminary studies have briefly reported the antiviral potential of HCQ; however, its specific dual regulatory mechanism against PRRSV has not been fully elucidated. Our observations are consistent with HCQ interfering with PRRSV‑triggered autophagic turnover, accompanied by LC3‑II accumulation and restored SQSTM1 protein abundance [32]. Notably, APP was screened as a top hub gene via computational network pharmacology, which may theoretically link HCQ activity to endosomal–lysosomal trafficking and autophagosome maturation. Nevertheless, no cellular or biochemical experiments were performed to validate functional crosstalk between HCQ and APP during PRRSV infection. This predicted regulatory relationship remains purely a computational hypothesis and cannot be validated using our current experimental dataset. Accordingly, we have tempered all mechanistic interpretations involving APP, and this lack of experimental validation is highlighted as one key limitation of this work. PRRSV preferentially infects porcine alveolar macrophages and hijacks autophagic signaling to amplify inflammatory responses, driving robust proinflammatory cytokine secretion and tissue injury [33, 34]. Consistent with network‑pharmacology predictions, IL‑6 was identified as another major hub bridging HCQ‑associated targets and inflammatory signaling. IL‑6 is a canonical NF‑κB‑dependent proinflammatory mediator capable of amplifying inflammation via autocrine and paracrine circuits. In our study, PRRSV infection enhanced IκBα phosphorylation and degradation, promoted NF‑κB p65 nuclear translocation, and upregulated IL‑6 expression; these molecular events were attenuated by HCQ in a concentration‑dependent fashion. These results reveal a functional association between the predicted IL‑6 hub and the NF‑κB cascade, suggesting that HCQ‑mediated suppression of NF‑κB may contribute to reduced IL‑6 levels and mitigation of PRRSV‑triggered inflammatory injury. Whether IL‑6 acts as a direct downstream effector mediating HCQ‑conferred protection still requires experimental verification. Collectively, network pharmacology proposes APP and IL‑6 as complementary regulatory nodes linking HCQ‑related targets to lysosomal autophagic flux and NF‑κB‑driven inflammation. Our experimental data align with this conceptual framework: HCQ altered autophagic processing while concurrently inhibiting NF‑κB activation and IL‑6 production. Although these correlations offer a working model for HCQ’s dual antiviral and antiinflammatory activities, direct APP‑dependent control of lysosomal autophagy and the exact contribution of IL‑6 to NF‑κB‑driven inflammation remain unproven.

Our in vivo piglet challenge model further validated HCQ-mediated protection against PRRSV-induced mortality, growth retardation, respiratory clinical signs, and pulmonary histopathological lesions and tissue viral load [35], alongside reduced secretion of inflammatory mediators, thereby alleviating the virus-triggered inflammatory response. Nevertheless, several limitations of the present study should be acknowledged when interpreting these findings. First, although our in vivo experiments demonstrated that HCQ provided protective effects against PRRSV infection, the in vivo efficacy of HCQ was evaluated only in 4-week-old nursery piglets under an experimental PRRSV challenge model. Therefore, whether these protective effects can be reproduced in pigs of different ages and physiological conditions, particularly in gestating sows, finishing pigs, and naturally infected commercial herds, remains to be determined. Second, although our cellular and in vivo data support the involvement of autophagy and NF-κB signaling in the protective effects of HCQ against PRRSV infection, the molecular mechanism underlying these effects has not been completely resolved. Our data demonstrate HCQ-associated changes in autophagic flux and NF-κB signaling, but we have not identified the direct molecular target(s) of HCQ among autophagy-related or signaling proteins, and no biochemical interaction data are available to establish a direct molecular interaction. In addition, the network pharmacology analysis identified candidate targets and signaling pathways potentially associated with the biological effects of HCQ, but these computational predictions have not yet been experimentally validated at the molecular level. Third, although the mCherry-EGFP-LC3 assay, together with LC3-II accumulation and SQSTM1 restoration, supports the conclusion that HCQ interferes with PRRSV-induced autophagic flux, the present study did not comprehensively dissect the individual molecular events underlying autophagosome–lysosome fusion and lysosomal function. Furthermore, genetic loss-of-function or rescue experiments targeting key autophagy-related factors were not performed, and therefore the causal contribution of autophagy inhibition to the antiviral effect of HCQ requires further validation. Fourth, although our in vitro experiments demonstrated that HCQ exhibited antiviral activity against genetically divergent PRRSV field isolates, PEDV, and SADS-CoV, these broad-spectrum in vitro effects were not evaluated in corresponding animal challenge models. Therefore, the current findings support the antiviral potential of HCQ against PEDV and SADS-CoV, but do not yet establish protective efficacy in vivo. Diverse swine RNA viruses may exploit conserved autophagy and endolysosomal pathways to support viral entry, intracellular trafficking, replication, or maturation, providing a potential common host dependency underlying the broad-spectrum activity of HCQ. Nevertheless, whether disruption of these pathways is sufficient to account for HCQ-mediated inhibition of PEDV and SADS-CoV remains unclear, as their specific autophagy requirements and downstream molecular mechanisms were not investigated in the present study. Fifth, although the present study identified effective antiviral concentrations of HCQ and demonstrated a favorable cytotoxicity profile in the tested cell models, systematic pharmacokinetic and pharmacodynamic evaluations were not performed. Therefore, the optimal therapeutic dose, dosing frequency, treatment duration, tissue exposure, and long-term safety profile of HCQ in pigs remain to be established. Finally, although our results support a coordinated role of autophagy and NF-κB signaling in the antiviral and antiinflammatory effects of HCQ, the complete regulatory network may involve additional uncharacterized signaling axes. In particular, the molecular crosstalk among autophagy, NF-κB signaling, oxidative stress, and inflammatory responses remains incompletely understood and requires further mechanistic investigation.

Future studies should focus on three complementary directions. First, genetic and biochemical assays are needed to identify direct molecular targets of HCQ and dissect causal relationships linking autophagy, NF‑κB signaling, oxidative stress, and PRRSV replication. Special attention should be paid to experimental validation of network‑pharmacology‑derived predictions such as the potential role of APP, which remains a purely computational finding in the current work. Second, animal challenge models are required to verify in vivo efficacy of HCQ against PEDV and SADS‑CoV and evaluate its translational value under field‑relevant conditions. Further work could also explore HCQ as an adjunct to commercial PRRS vaccines, assessing impacts on viral replication, vaccine‑elicited humoral and cellular immunity, and overall protective potency. Follow‑up field investigations will be essential to evaluate whether HCQ‑based interventions (alone or combined with vaccination) can reduce viral shedding and disease burden, while maintaining satisfactory safety, growth performance and economic feasibility on commercial pig farms. Collectively, these efforts will clarify whether HCQ’s antiviral properties observed under experimental settings can be translated into scalable practical control strategies for PRRSV and other swine viral pathogens.

## Data Availability

The data used in the network pharmacology analysis of Hydroxychloroquine (HCQ) against PRRSV in this study were obtained from the following public databases—HCQ target data: derived from the STITCH database and the DrugBank database.—PRRSV-related host target data: derived from the GeneCards database and the DisGeNET database.—Protein–protein interaction (PPI) data: derived from the STRING database.—GO/KEGG enrichment analysis data: derived from the Metascape database. All processed data supporting the findings of this study are presented in the main text and figures of this article, and are available to readers through this article.

## Declarations

### Declaration of generative artificial intelligence (AI) and AI-assisted technologies

During the preparation of this manuscript, the authors used AI writing tools for language polishing. All content was fully reviewed, revised and verified by all authors, who take full responsibility for all data and conclusions in this work.
